# Molecular epidemiology of hydropericardium syndrome outbreak-associated serotype 4 fowl adenovirus isolates in central China

**DOI:** 10.1186/s12985-016-0644-x

**Published:** 2016-11-18

**Authors:** Teng Zhang, Qianyue Jin, Peiyang Ding, Yinbiao Wang, Yongxiao Chai, Yafei Li, Xiao Liu, Jun Luo, Gaiping Zhang

**Affiliations:** 1 College of Life Science, Henan Agricultural University, Wenhua Road No. 95, Zhengzhou, 450002 People’s Republic of China; 2 Key Laboratory of Animal Immunology of the Ministry of Agriculture, Henan Provincial Key Laboratory of Animal Immunology, Henan Academy of Agricultural Sciences, Huayuan Road No.116, Zhengzhou, 450002 People’s Republic of China; 3 College of Animal Husbandry and Veterinary Medicine, Henan Agricultural University, Zhengzhou, 450002 People’s Republic of China; 4 College of Veterinary Medicine, Northwest Agriculture and Forestry University, Yangling, 712100 Shaanxi China; 5 School of Public Health, Xinxiang Medical University, Xinxiang, 453003 People’s Republic of China; 6 Jiangsu Co-innovation Center for Prevention and Control of Important Animal Infectious Diseases and Zoonoses, Yangzhou, 225009 People’s Republic of China

**Keywords:** Hydropericardium syndrome, Chicken, Molecular epidemiology, Serotype 4 fowl adenovirus

## Abstract

In several parts of China, there have been a large number of hydropericardium syndrome (HPS) outbreaks caused by serotype 4 fowl adenovirus (FAdV­4) in broiler chickens since 2015. These outbreak-associated FAdV-4 strains were distinct from previous circulating strains which did not lead to severe HPS outbreaks. To better understand the molecular epidemiology of the currently circulating FAdV strains for effective diagnosis and treatment of HPS, we isolated 12 HPS outbreak-associated FAdV-4 strains from different regions in central China and investigated their molecular characteristics by performing phylogenetic analyses based on the hexon genes. Our results indicated the FAdV-4 strains in this study all belonged to serotype FAdV-4, species FAdV-C. And in comparison with ON1, KR5, MX-SHP95, PK-01, PJ-06 strains within the cluster where outbreak-associated FAdV-4 strains were located, the nucleotide sequence divergence were 1.31, 1.10, 1.42, 2.77 and 2.84%, respectively. Phylogenetic analyses revealed the hexon genes of the 12 outbreak-associated strains clustered to a relatively independent branch of the tree, and evolved from the same ancestor and we suggested that these outbreak-associated FAdV-4 strains originate from earlier strains in India.

## Introduction

Hydropericardium syndrome (HPS) is an infectious viral disease in broiler birds at 3 to 5 weeks of age. It is caused by serotype 4 fowl adenovirus (FAdV-4) and characterized by hydropericardium and hepatic necrosis [1]. HPS was also known as “Angara Diesase”, because its first outbreak was observed in the Angara Goth, Pakistan in 1987 [2, 3]. Until now, HPS has been reported in many countries including Iraq [1], Kuwait, India [4], Mexico, Ecuador, Peru, Chile [5], USA [6], Russia [7], Japan [8, 9], and Poland [10], resulting in considerable economic losses.

Fowl adenoviruses (FAdVs) are non-enveloped double stranded DNA-viruses and belong to the genus Aviadenovirus, family Adenovirida together with other four genera: Mastadenovirus, Atadenovirus, Siadenovirus, and Ichtadenovirus [11]. Based on restriction enzyme digest pattern and serum cross-neutralization test, FAdVs have been grouped into 5 species (FAdV-A to FAdV-E) with 12 serotypes (FAdV-1 to 8a and 8b to 11) [12]. Serotype 4 fowl adenovirus (FAdV-4), the causative agent of HPS, is a member of the species Fowl Adenovirus C [4, 13]. The genome of FAdV-4 encodes a number of non-structural proteins and three structural proteins: hexon, penton and the fiber protein. The hexon gene of FAdVs is the longest and consists of hypervariable Loop L1 (HVR1-4) regions, making it a hotspot for research on taxonomy and antigenic shift of FAdVs [10, 14–16]. Hexon protein is the predominant target for induction of serotype-specific neutralizing antibodies [13].

**Fig. 1:**
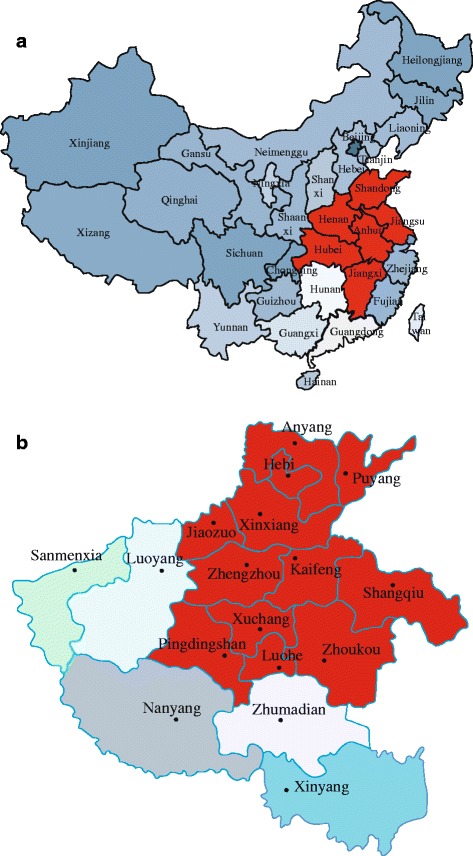
Distribution of HPS outbreaks in China (**a**) and in Henan province (**b**). Since July 2015, HPS outbreaks have been reported in Henan, Shangdong, Anhui, Jiangxi, Jiangsu, and Hubei provinces of China, which causes huge losses and continues to threaten the poultry industry (**a**). In this study, 12 FAdV field strains from 12 regions in central China were isolated in HPS-outbreak chicken flocks and propagated in CEF cells (**b**)

Since 2015, clinical cases of HPS have been increasing in many regions of China, including Shandong, Hubei, Jiangsu, Anhui, Jiangxi, and Henan (Fig. 1a). These outbreaks were characterized by high mortality and no seasonal characteristics and mainly concentrated in small and medium broiler farms, rearing chickens and ducks [17]. However, to date, little is known regarding the molecular and genetic evolution characteristics of these potentially devastating FAdV strains that remain circulating in China. Hence, in this study, we aimed to investigate the molecular epidemiology of these currently circulating strains in chicken flocks in central China. Phylogenetic trees were constructed based on the hexon genes to establish the origin and genetic relationships of FAdV strains. It was found that FAdV field strains circulating after 2015 were closely related to the Indian strains PK-01 and PJ-06.

## Materials and methods

### Origin of the strains

Twelve liver samples of chickens were collected from flocks with HPS outbreaks in 12 different regions of Henan, central China, and frozen at −20 °C. All samples were confirmed to be FAdV-4 positive by polymerase chain reaction (PCR) amplifying a 632-bp fragment (Named fragment I) with primers (Table 1) based on the polymerase gene of FAdV strain MX-SHP95 (GenBank No. KP295475.1). Reactions were performed according to the following protocol: 95 °C for 5 min, followed by 31 cycles of 95 °C for 30 s, 56 °C for 30 s, 72 °C for 50 s, and a final elongation step of 10 min at 72 °C. 24 reference FAdV strains and 12 outbreak-associated FAdV strains used for phylogenetic analyses were listed in Table 2.

**Table 1:** Primers used in the PCRs

Target genes	Primers	Expected product
Fragment I	F: GCAGCGTGGTCTTGAAGATGGTTC	632 bp
	R: CGCATTCAAGCCCGTTCGATTC	
Fragment A	F: CGTCTAGGTTCGCACCGCCATGGC	1501 bp
	R: CATCTGGTCGATGGACCAACGCGCACC	
Fragment B	F: CATCGACCAGATGGACAACGTCAACCCCTTCAAC	1345 bp
	R: TTACACGGCGTTGCCTGTGGCG	

All samples were confirmed to be FAdV-4 positive by PCR amplifying a 632-bp fragment (Named fragment I) of the polymerase gene. The hexon gene of FAdV-4 was divided into two fragments (Named fragment A and B) because of its long length in this study. Primers used for amplifying fragments I, A and B were designed according to the polymerase genes and hexon genes of FAdV-4 strain MX-SHP95 (GenBank No. KP295475.1) and synthesized by Sangon Biotech, Shanghai, China

**Table 2:** FAdV strains and isolates used for sequence alignment and phylogenetic analysis

Species	Strains and serotypes	Accession no.	Origin
FAdV-A	FAdV-1	U46933.1 (complete genes)	Germany
	FAdV-1 CELO	Z67970.1 (complete genes)	Russia
FAdV-B	FAdV-5 340	KC493646.1 (complete genes)	--
	FAdV-5 TR22	AF508953.1 (partial cds)	--
	FAdV-B 09-7473-2	FN869988.1 (partial cds)	--
FAdV-C	FAdV-4 KR5	HE608152.1 (complete genes)	Austria 2012
	FAdV-4 HB1510	KU587519.1 (complete genes)	China 2015
	FAdV-4 HN151025	KU245540.1 (complete genes)	China 2015
	FAdV-4 JSJ13	KM096544.1 (complete genes)	China 2015
	FAdV-4 Kr-Yeoju	HQ709228.1 (complete genes)	Korea 2011
	FAdV-4 Kr-Gunwi	HQ709227.1 (complete genes)	Korea 2011
	FAdV-4 ON1	GU188428.1 (complete genes)	China 2011
	FAdV-4 PJ-06	EU931692.1 (complete genes)	India 2008
	FAdV-4 PK-01	EU931693.1 (complete genes)	India 2008
	FAdV-4 MX-SHP95	KP295475.1 (complete genes)	Mexico 2015
	FAdV-4 HNZZ	KX640901 (complete genes)	China 2016
	FAdV-4 HNAY	KX640902 (complete genes)	China 2016
	FAdV-4 HB	KX640903 (complete genes)	China 2016
	FAdV-4 JZ	KX640904 (complete genes)	China 2016
	FAdV-4 XX	KX640905 (complete genes)	China 2016
	FAdV-4 XC	KX640906 (complete genes)	China 2016
	FAdV-4 LH	KX640907 (complete genes)	China 2016
	FAdV-4 PY	KX640908 (complete genes)	China 2016
	FAdV-4 KF	KX640909 (complete genes)	China 2016
	FAdV-4 SQ	KX640910 (complete genes)	China 2016
	FAdV-4 ZK	KX640911 (complete genes)	China 2016
	FAdV-4 PDS	KX640912 (complete genes)	China 2016
	FAdV-10	U26221 (complete genes)	US 2000
FAdV-D	FAdV-2 SR48	AF508946.1 (partial cds)	--
	FAdV-3 SR49	AF508948.2 (partial cds)	--
	FAdV-11 C2B	AF508959.2 (partial cds)	US
	FAdV-D	AC_000013.1 (complete genes)	--
FAdV-E	FAdV-6 CR119	AF508954.2 (partial cds)	Japan
	FAdV-7 YR36	AF508955.1 (partial cds)	Japan
	FAdV-8 58	AF508957.1 (partial cds)	--
	FAdV-8a TR59	AF508956.2 (partial cds)	Japan

Twelve strains were isolated and confirmed to be FAdV-4 positive by PCR. Sequenced Hexon genes from these strains were edited using DNAStar software, and deposited into GenBank under the accession number KX640901-KX640912. 24 strains from GenBank were used as reference strains. Serotypes, species and origins of these strains were provided. “--” indicated unknown

### Genomic DNA extraction

The presence of FAdV in each sample was confirmed by virus isolation followed by PCR. Initially, 5 mg of liver was cut into tiny pieces and fully ground in sterilized PBS (weight/volume = 1:3) under aseptic conditions. Following complete grinding, the suspension of liver tissue in PBS was frozen at −20 °C and thawed at 37 °C three times before centrifugation at 8000 rpm at 4 °C for 10 min. The supernatant was then collected, filtered through a 0.22 μm filter, and inoculated onto a confluent monolayer of CEF (chicken embryo fibroblast) cells prepared from 9-day SPF embryonated chicken eggs. After incubation for 1 h at 37 °C, 1.5 ml of DMEM media containing 2% fetal bovine serum, penicillin (100 U/ml), and streptomycin (100 μg/ml) were added to the 6-well cell culture plate. 72 h later, supernatants were collected and filtered through a 0.22 μm filter for DNA extraction. Genomic DNA from these field samples was extracted using TaKaRa MiniBEST Universal Genomic DNA Extraction Kit Ver.5.0 (TaKaRa, Dalian, China), and stored at −20 °C before use. Quantification and quality of DNA extracts were determined with a Nanodrop spectrophotometer (Thermo Scientific, USA).

### PCR

The hexon gene was divided into two fragments (Named fragment A and B) because of its long length in this study. Primers used for amplifying fragments A and B were designed according to the hexon genes of FAdV-4 strain MX-SHP95 (GenBank No. KP295475.1) and synthesized by Sangon Biotech (Table 1). PCR was performed in a Bio-Rad MyCycler Thermal Cycler. Fragments A and B were amplified in a 25 μl PCR reaction containing 1.5 μl genomic DNA (200–400 ng/μl), 0.25 μl Q_5_ High-Fidelity DNA Polymerase, 0.5 μl dNTPs (10 mM), 1.25 μl forward primer (10 pmol/μl), 1.25 μl reverse primer (10 pmol/μl), 10.25 μl sterilized water, 5 μl 5X Q_5_ Reaction Buffer, and 5 μl 5X Q5 High GC Enhancer. Two expected size of fragment A (1501 bp) and fragment B (1345 bp) were amplified using the following conditions: initial denaturation at 98 °C for 3 min; 31 cycles of denaturation at 98 °C for 10 s, annealing at 66 °C (for fragment A) and 57 °C (for fragment B) for 30s, extension at 72 °C for 80s; and final extension at 72 °C for 5 min. PCR products for each sample were visualized by electrophoresis in a 1.0% agarose gel containing ethidium bromide, purified according to the manufacturer’s protocol, using Universal DNA Purification Kit (TIANGEN, Beijing), and ligated into pEASY-Blunt Cloning vector using pEASY-Blunt Cloning Kit (TransGen Biotech) according to the manufacturer’s protocol. Upon transformation of each ligation reaction mixture into Trans1-T1 Phage Resistant Chemically Competent cells, a single colony was picked up and identified by PCR and restriction enzyme digestion before sequencing.

### Sequencing and phylogenetic analysis

The respective bacterial clones harboring recombinant plasmids of fragment A and fragment B were sequenced by Sangon Biotech. Each sample was sequenced twice (one with the primers designed in this study and the other with the sequencing primers of the PEASY-Blunt Cloning Vector). The obtained sequences were edited using DNAStar software, and deposited into GenBank. Homology analyses to identify gene homologs were performed with the BLAST program (NCBI). Phylogenetic trees were constructed using MEGA software by the neighbor-joining analysis with 1000 bootstrap replicates, Maximum Composite Likelihood method. The phylogenetic datasets for analyses included 12 FAdV-4 isolates from this study and 24 reference strains (Table 2).

## Results

Before 2014, FAdV-4 circulation also existed in China, such as ON1 (2004), JSJ13 (2013), but there were no significant HPS outbreaks. However, since July 2015, HPS outbreaks have been reported in Henan, Shandong, Anhui, Jiangxi, Jiangsu, and Hubei provinces of China (Fig. 1a), which causes huge losses and continues to threaten the poultry industry. In this study, 12 FAdV field strains from different regions in central China (Fig. 1b) were isolated in HPS-outbreak chicken flocks and propagated in CEF cells. After PCR amplification and sequencing of the hexon genes, the obtained complete hexon gene sequences were deposited into GenBank under the following accession numbers: KX640901- KX640912 (Table 2).

**Fig. 2:**
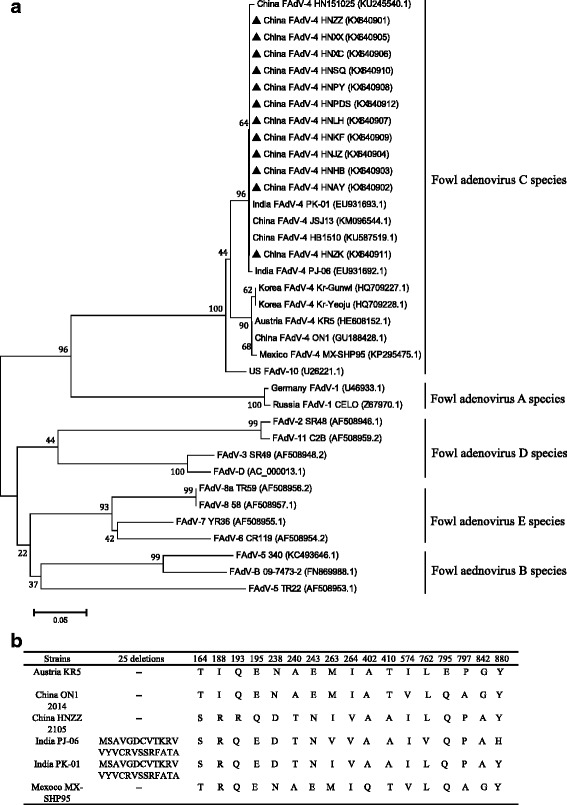
Phylogenetic analysis of nucleotide sequences of the Hexon genes (**a**) and Alignment of amino acid sequences (**b**). Phylogenetic trees were constructed using MEGA software by the neighbor-joining analysis with 1000 bootstrap replicates, Maximum Composite Likelihood method. The phylogenetic datasets for analyses included 12 FAdV-4 isolates from this study (*filled triangles*) and 24 reference strains (**a**). And alignment of amino acid sequences including those FAdV-4 strains: KR5, ON1, HNZZ (on behalf of the strains in this study), PJ-06, PK-01, MX-SHP95

Hexon genes were used to analyze whether changes in hexon protein could explain the sudden outbreak. A phylogenetic tree based on the hexon protein included 12 outbreak-associated FAdV-4 strains from this study and 24 strains from GenBank (Table 2) produced four distinct groups: the first formed by FAdV-A (U46933.1, Z67970.1), the second formed by FAdV-C (KR5, HB1510, HN151025, ON1, JSJ13, PJ-06, PK-01, Kr-Gunwi, Kr-Yeoju, MX-SHP95, FAdV-10) and the 12 strains isolated in this study; the third formed by FAdV-D (SR48, SR49, C2B, FAdV-D), and FAdV-E (CR119, YR36, 58, TR59), and the last formed by FAdV-B (340, TR22, 09-7473-2) (Fig. 2a).

The hexon genes in this study shared 100% identity both in the nucleotide and amino acid level with FAdV-4 HB1510 (China, 2015) and FAdV-4 SSDX (China, 2015), which clustered in the same branch with JSJ13 (China, 2013), PK-01and PJ-06 (India, 2008). In comparison with the hexon genes in this study, ON1, KR5, MX-SHP95, JSJ13, PK-01, PJ-06 strains within the cluster, displayed variability of 1.31, 1.10, 1.42, 25.16, 2.77 and 2.84 %, respectively at the nucleotide level. Though before 2014 FAdV-4 strain ON1 (2004, China) and JSJ13 (2011, China) had circulated in China, there were no significant HPS outbreaks. Compared with the hexon genes in this study, JSJ13 contained a deletion of 708 bases at the 5′ end albeit the rest is in full agreement, which may help explain the scare outbreaks of HPS before 2014. And alignment of amino acid sequences revealed that compared with ON1, all of the 12 outbreak-associated FAdV-4 strains contain 37 bases substitutions and as many as 13 amino acid substitutions including T to S at position 164, I to R at position 188, Q to R at position 193, E to Q to at position 195, N to D at position 238, A to T at position 240, E to N at position 243, M to I at position 263, I to V at position 264, T to A at position 410, V to I at position 574, A to P at position 797, G to A at position 842 (Fig. 2b). These mutations may help to explain the sudden outbreaks.

However, compared with Indian strains PK-01 and PJ-06, the hexon proteins only contain 2, 5 amino acid substitutions respectively, despite of 25 amino acid deletions at the N end, which do not lead to decline of the virulence. And the genetic relationship between them is very close. Thus, we postulate that ancestors of the strains is not the local strains but earlier strains in India.

## Discussion

FAdVs are commonly present in chicken farms worldwide [2]. HPS is the most severe disease associated with FAdV infection, which has been attributed exclusively to serotype 4. Before 2014, FAdV-4 circulation had been reported in China, but there were no severe HPS outbreaks. However, since July 2015, outbreaks of HPS occurred with sudden high mortality rates in broilers in many small and middle chicken forms in central China, even the duck farms, leading to tremendous economic losses. Recent years, the frequency of HPS have also been increasing in many countries, such as India (2014) [18], Canada (2011) [19], Hungary (2013) [20], Korea (2012) [21], Japan (2012) [22], and Poland (2016) [10]. The ON1 and JSJ13 strains in local regions in China didn’t lead to severe clinical symptoms. The phylogenetic tree revealed that the strains circulated in China before 2014 and after July 2015 had different ancestors, which lead to this phenomenon. And the strains circulated in China now derived from earlier strains in India, with some mutations and 25 amino acid deletions; however, the exact role of the deletion in these FAdVs still needs to be elucidated in future studies.

Recently, it has been demonstrated that FAdV-4 strains circulating in China carried deletion within ORF19, which might lead to higher virulence [3]. And in comparison with JSJ13 and JH13 (isolated from China in 2013), the strains HB1510 (isolated from China in 2015) and MX-SHP95 (isolated from Mexico), KR-5 (isolated from Australia) carried a deletion of 33 nt in ORF29 [3]. This indicated that FAdV strains also carried changes in other genes and the exact of these changes in relation to antigenic variation and pathogenicity should be further investigated.

Currently, commercial vaccines against HPS have not yet been developed in China due to the lack of knowledge and understanding of FAdVs. Shengwang Liu and Huixin Li also confirmed that HPS outbreak is attributed to the emergence of FAdv strains in chickens in China, and what’s worse, FAdV species C, D, and E were co-circulating in chicken flocks in China, which made the infection more complex [23]. The molecular and phylogenetic analysis based on hexon genes in this study may further the understanding of FAdV evolution and provide relevant information for developing vaccines against the disease.

## Conclusions

FAdV-4 strins is continuously affecting the poultry industry of China all over the world. In this study, we investigated the molecular epidemiology and characteristics of HPS outbreak-associated FAdV-4 strains based on the hexon genes. It showed that FAdV strains isolated in central China in this study all belonged to serotype FAdV-4, species FAdV-C. Besides, FAdV-4 strains (represented by FAdV ON1) which did not cause many HPS outbreaks before 2015 cluster together with the Austria strain KR5, Mexico strain MX-SHP95. FAdV strains now circulating in central China since 2015 are closely related to the Indian strains PK-01 and PJ-06, which revealed that the strains now originated from the earlier strains in India. This study also provides new information about the prevalence of those FAdV-4 strains currently circulating in central China, which contributes to the prevention and contral, even elimination of the strains.

## Abbreviations

BLAST: Basic local alignment search tool
CEF: Chicken embryo fibroblast cells
FAdV-4: Serotype 4 fowl adenovirus
FAdVs: Fowl adenovirus
HPS: Hydropericardium syndrome
HVR: Hypervariable loop
PCR: Polymerase chain reaction
SPF: Specific pathogen free
